# *Cytomegalovirus*, a “Friend” of SARS-CoV-2: A Case Report

**DOI:** 10.3390/children11081010

**Published:** 2024-08-19

**Authors:** Nicoleta-Ana Tomşa, Lorena Elena Meliţ, Gabriela Bucur, Anca-Meda Văsieșiu, Cristina Oana Mărginean

**Affiliations:** 1 Pediatrics Clinic, Emergency Clinical County Hospital, 540140 Targu Mures, Romania; tomsa.nicoleta@yahoo.com (N.-A.T.); gabriela.bucur@spital.mures.ro (G.B.); 2 Department of Pediatrics 1, George Emil Palade University of Medicine, Pharmacy, Science and Technology of Targu Mures, 540136 Targu Mures, Romania; oana.marginean@umfst.ro; 3 Department of Infectious Disease, George Emil Palade University of Medicine, Pharmacy, Science, and Technology of Targu Mures, 540136 Targu Mures, Romania; anca-meda.vasiesiu@umfst.ro

**Keywords:** MIS-C, CMV, SARS-CoV-2, child, pneumonia

## Abstract

Introduction: Cytomegalovirus (CMV) infection is present in a latent state in 70–90% of the immunocompetent population, and its reactivation might be triggered by inflammatory conditions such as post-COVID multisystem inflammatory syndrome (MIS-C) or by immunosuppression induced by steroids. The aim of this paper was to highlight the unexpected complications associated with SARS-CoV-2 infection that require a complex clinical approach for accurate diagnosis. Materials and Methods: We present the case of a 4-year-old male patient who, during an initially favorable course of PIMS, experienced symptoms of respiratory failure. Results: The patient initially presented with clinical and paraclinical signs of PIMS with cardiac involvement, for which high-dose corticosteroid therapy was initiated, followed by gradual tapering, along with immunoglobulins, anticoagulants, antiplatelet agents, and symptomatic treatment. After 10 days of favorable progress, the patient’s general condition deteriorated, showing tachypnea, desaturation, and a ground-glass appearance on thoracic CT. Negative inflammatory markers and favorable cardiac lesion evolution ruled out MIS-C relapse. The presence of anti-CMV IgM antibodies and viral DNA in the blood confirmed acute CMV infection, likely triggered by prior severe-acute-respiratory-syndrome-related coronavirus 2 (SARS-CoV-2) infection and secondary immunosuppression due to steroids. Non-specific immunomodulatory treatment was initiated but led to worsening of pulmonary lesions, prompting the initiation of specific antiviral treatment with ganciclovir, resulting in rapid clinical and imaging improvement. Conclusions: CMV infection can be reactivated by immunosuppression induced by corticosteroid therapy for MIS-C and may require specific etiological treatment.

## 1. Introduction

Human cytomegalovirus (CMV), also known as human herpesvirus 5, is a member of the Betaherpesvirinae subfamily within the Herpesviridae family. It is a double-stranded DNA virus that typically causes primary infection during early childhood and remains latent in the host’s white blood cells [1,2]. In immunocompetent children, primary CMV infection usually results in a mild illness characterized by lymphopenia, lymphadenopathy, fever, and hepatosplenomegaly, and it can sometimes be asymptomatic. The major concern is related to children with primary or secondary immunodeficiency due to various causes, including immunosuppressive treatments, corticosteroid therapy, and hematopoietic or organ transplantation [1,3]. Also, infection and reactivation of CMV are frequently encountered in patients with advanced HIV disease and have significant implications for the progression of the underlying disease [4].

The gastrointestinal tract is frequently affected by CMV infection. CMV gastrointestinal disease is characterized by clinical symptoms related to the gastrointestinal system, observable endoscopic findings, and detection of CMV in tissue through methods such as histopathology, culture, immunohistochemistry (IHC), or DNA hybridization techniques. Studies, including case reports and case series, have shown that CMV gastrointestinal disease can present in diverse ways and be managed with a range of treatments, particularly in immunocompetent individuals [5].

The most commonly used antiviral therapy for immunocompromised populations, including pediatric patients, is intravenous ganciclovir, a synthetic guanine nucleoside, administered intravenously due to its very poor oral bioavailability [6]. Ganciclovir has significant cellular toxicity, with major side effects including neutropenia, anemia, thrombocytopenia, diarrhea, and fever. This therapy is used for treating CMV infection in patients with AIDS or other immunocompromised conditions, as well as in cases of CMV-confirmed pneumonia or post bone marrow transplant. Another treatment option is valganciclovir, a prodrug of ganciclovir, which is well absorbed in the gastrointestinal tract of children and rapidly metabolized into ganciclovir in the intestinal wall and liver, with a bioavailability of 60%. The adverse effects are similar, but the main advantage of valganciclovir is its higher bioavailability. Other agents used in CMV infection include foscarnet, cidofovir, maribavir, or specific immunoglobulin [7,8].

Severe-acute-respiratory-syndrome-related coronavirus 2 (SARS-CoV-2) first appeared in Wuhan, China, in December 2019 and rapidly spread to Europe, particularly the northern regions of Italy, in early February 2020 [9]. Initial reports from China indicated that pediatric populations exhibited milder symptoms compared to adults, suggesting several theories: differences in immune systems, overall better health status, and cross-reactive immunity from common cold viruses. However, the statement that COVID-19 infection/illness in children is always mild has been further challenged by reports of children presenting with a severe, albeit infrequent, inflammatory multisystem syndrome from the United Kingdom, Italy, Spain, and New York [9,10]. These case series have observed symptoms resembling Kawasaki-like syndrome: high fever, conjunctivitis, lymphadenopathy, skin rashes, and coronary artery involvement (dilation in severe cases), along with cardiovascular shock, encephalitis, and multiple organ failure [10,11]. Thus, it has been reported that these symptoms occur 2–4 weeks after SARS-CoV-2 infection, and the condition was called multisystem inflammatory syndrome post-COVID (MIS-C). The precise causes of this condition are still not fully understood but are explained as a hyperreactivity of the immune system [12,13]. The diagnostic criteria were developed in 2020 by the Royal College of Paediatrics and Child Health in the UK, the US Centers for Disease Control and Prevention, and the World Health Organization, which also proposed the name for this condition: multisystem inflammatory syndrome in children (MIS-C) [14,15,16].

The goal of MIS-C treatment is to stabilize the patient, manage life-threatening symptoms, and prevent long-term sequelae, especially cardiac complications such as coronary artery aneurysms, myocardial fibrosis, and rhythm or conduction disorders that may occur [17,18]. The first-line anti-inflammatory therapy for all patients with MIS-C is intravenous immunoglobulin (IVIG), with second-line therapy being intravenous methylprednisolone for children who remain symptomatic after 24 h of first-line treatment. Biologic therapy should be considered as a third-line option [18]. Patients with MIS-C and prothrombotic risk factors may benefit from prophylactic anticoagulant therapy, typically using enoxaparin [19]. Treatment with aspirin is usually necessary for 1–4 weeks, with the possibility of extension up to 12 weeks. Anti-inflammatory therapy should be continued until all symptoms resolve, and normalization of inflammatory markers such as erythrocyte sedimentation rate (ESR) and C-reactive protein (CRP) is considered indicative of resolution of the inflammatory syndrome [20]. We must mention that with the lack of treatment, this condition can be fatal.

The aim of this case report was to highlight the possibility of unexpected, rare complications associated with SARS-CoV-2 infection that require a complex clinical approach for accurate diagnosis.

## 2. Case Presentation

### 2.1. History and Reasons for Admission

We present the case of a 4-year-old male with a history of recurrent upper respiratory tract infections and SARS-CoV-2 infection based on a positive rapid antigen test approximately 5 weeks prior to this admission. He was admitted in November 2021 for the following symptoms: high fever (up to 40 °C), serous rhinorrhea, abdominal pain, diarrhea, vomiting, decreased appetite, and myalgia, with the onset 4 days prior to this admission. The patient had received antibiotic amoxicillin with clavulanic acid in a dose of 40 mg per kg of body weight per day for 3 days and symptomatic treatment at home without improvement.

### 2.2. Physical Examination

Upon admission, the clinical examination revealed the following: influenced general status, pallor, macular rash on the anterior and posterior chest, scrotal hyperemia, hyperemic and hypertrophic tonsils, hyperemic conjunctiva, bilateral cervical enlarged lymph nodes, tachycardia (150 beats/min), blood pressure of 101/64 mmHg, abdominal tenderness, accelerated bowel sounds, weight 22 kg.

### 2.3. Diagnostic Elements and Evaluation

Laboratory tests revealed the following: neutrophilia (9160 × 10^3^/μL, 80%), thrombocytopenia (123,000 × 10^3^/μL), mildly decreased hemoglobin (10.2 g/dL) and serum albumin (3.51 g/dL), markedly elevated inflammatory markers (erythrocyte sedimentation rate—ESR: 98 mm/h; C-reactive protein—CRP > 180 mg/L), ferritin (550.8 ng/mL), and D-dimer levels (731 ng/mL). The evaluation of the cardiac parameters underlined elevated CK-MB (40.9 U/L), NT-proBNP (22,317 pg/mL), and Troponin I (119 ng/L). Seriated blood cultures in the setting of fever or its absence were negative. The echocardiography highlighted moderate systolic–diastolic dysfunction with left coronary artery dilation. Based on the history (SARS-CoV-2 infection approximately 5 weeks prior to the admission), the clinical presentation (high fever, gastrointestinal symptoms, skin involvement), and the paraclinical findings (elevated inflammatory markers, abnormal coagulation tests, cardiovascular impairment), we established the diagnosis of MIS-C. We must mention that due to objective reasons, we did not perform the SARS-CoV-2 IgG antibodies at the time of admission (lack of appropriate tests in the laboratory).

Thus, we initiated immunomodulatory treatment with intravenous immunoglobulin (2 g/kg/24 h), but because the patient remained symptomatic after immunoglobulin administration, we decided to associate high-dose corticosteroid therapy (pulsed therapy, 20 mg/kg/day for 3 days, followed by gradual tapering), antiplatelet therapy, anticoagulant therapy initiation after platelet count normalization, and supportive cardiovascular therapy (Lisinopril, Spironolactone).

### 2.4. Therapeutic Management, Monitoring, and Evolution

**Figure 1:**
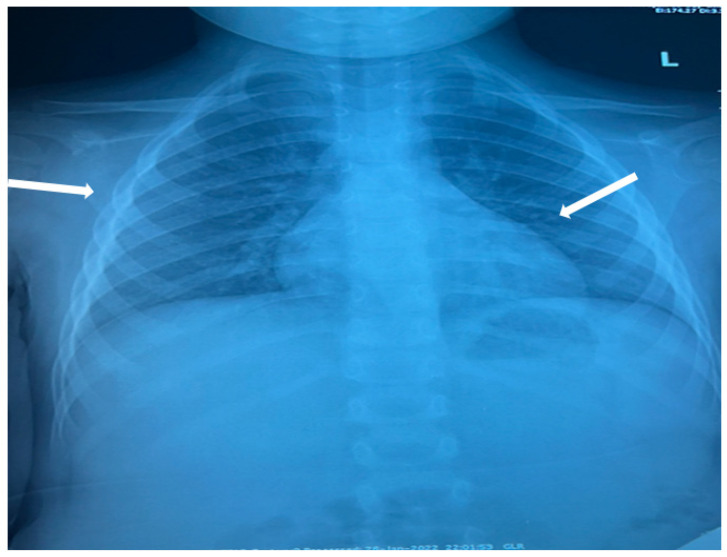
Diffuse peribronchovascular infiltrates and inferior interclavicular hilar infiltrates (white arrows).

**Figure 2:**
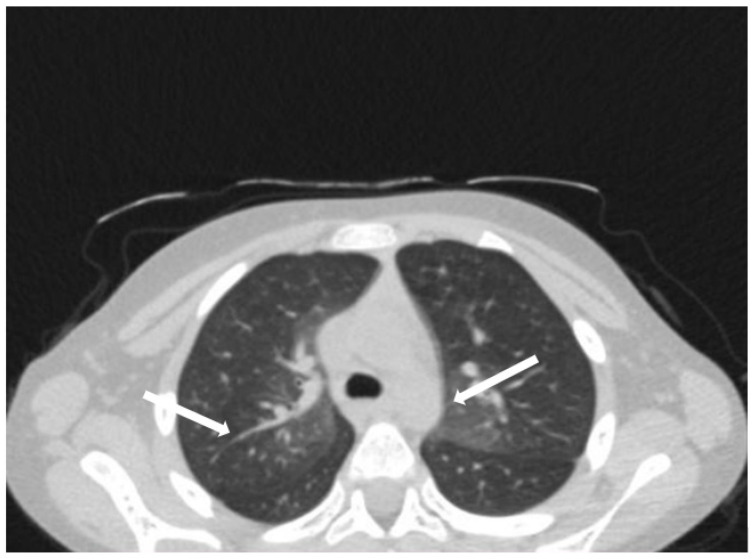
Ground-glass opacities with subpleural disposition adjacent to the mediastinum, in the upper lobes at the apical and posterior segments of the right lung, and at the apicoposterior segment of the lung (white arrows).

**Figure 3:**
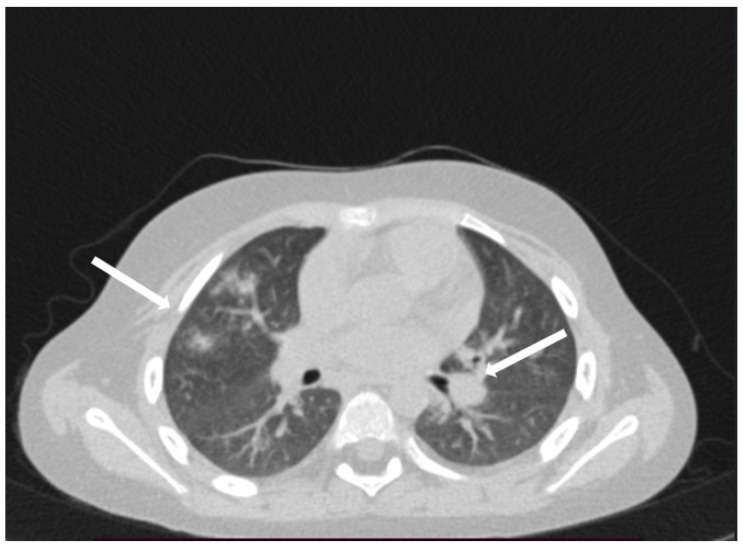
In the lower pulmonary lobes, opacities were observed in the bilateral posterior segments: left anteromedial and right medial segments (white arrows).

The patient was hospitalized in the Pediatric Clinic I for 3 weeks, being closely monitored (seriated clinical exam, laboratory tests, and echocardiography). The patient’s evolution gradually became favorable, and he was discharged with the recommendation to continue antiplatelet therapy and low-dose oral steroids. However, 4 days after discharge, the patient presented to the Emergency Department with altered general condition, respiratory distress, and fatigue. Laboratory tests revealed leukocytosis (27.720 × 10^3^/μL), with neutrophilia (22.970 × 10^3^/μL), thrombocytosis (491 × 10^3^/μL), monocytosis (2.180 × 10^3^/μL), and elevated inflammatory markers (CRP > 180 mg/L, ESR 43 mm/h), while urine analysis and cardiac enzymes were within normal limits. In the emergency department, a chest X-ray was performed which pointed out diffuse peribronchovascular infiltrates and inferior interclavicular hilar infiltrates (Figure 1). Given the influenced general condition and the presence of respiratory failure (oxygen saturation of 90%), along with the findings of the chest X-ray, we decided also to perform pulmonary computed tomography (CT), which identified ground-glass opacities with subpleural disposition adjacent to the mediastinum, in the upper lobes at the apical and posterior segments of the right lung, and at the apicoposterior segment of the left lung. Additionally, in the lower pulmonary lobes, opacities were observed in the bilateral posterior segments, left anteromedial, and right medial segments, indicative of possible pulmonary hypertension/incipient pulmonary edema/acute respiratory distress syndrome (ARDS) (Figure 2 and Figure 3). Pulmonary hypertension and pulmonary edema were ruled out based on clinical findings, cardiac examination, and normal cardiac enzymes. Based on all these findings, we considered pneumonia in the context of immunosuppression and we performed several tests to identify the possible etiology, finding elevated positive anti-CMV IgM antibodies and increased IgG antibodies of CMV (967.9 U/mL), which were confirmed by subsequent repeated tests. Additionally, we found evidence of hepatocellular injury (ALT: 166 U/L; AST: 60.7 U/L) prompting intravenous hepatoprotective treatment and initiation of an unspecified immunomodulatory agent, inosine acedoben dimepranol, orally, at a dose of 50 mg/kg body weight per day. This approach was chosen due to limitations in specific antiviral therapy for the pediatric population.

With the aforementioned treatment, initially, the evolution was moderately favorable, but after approximately 3 days, desaturations recurred to 89–90% in ambient air, prompting the decision to perform CMV viremia testing, which was tested via the polymerase chain reaction (PCR) method, which is the most common and sensitive method; quantitative PCR can measure the viral load by amplifying CMV DNA in the blood sample. The result returned positive (CMV DNA 198 IU/mL). Therefore, we initiated specific antiviral therapy, following a thorough explanation of potential side effects, using intravenous ganciclovir in dose of 5 mg/kg body weight per dose at 12 h intervals, administered for 14 days. After 5 days of antiviral therapy, the patient presented a clinical improvement with an overall better general condition, and by days 7–8 of treatment, oxygen therapy was no longer needed. CMV viremia, on day 10 after treatment, showed undetectable levels. Subsequent progress at 2 weeks, monthly for 6 months, and then annually, showed good recovery without the recurrence of respiratory or other events.

The patient’s mother’s informed consent was obtained prior to the publication of this case.

## 3. Discussion

MIS-C is a relatively new entity, often mistaken for Kawasaki disease. The distinguishing factor is the recent history of SARS-CoV-2 infection and the presence of IgG antibodies anti-SARS-CoV-2 in the serum [21]. On the other hand, CMV infection is widespread, with over 4 billion people infected globally. While mostly congenital, CMV infection can occur at any time during life and may be asymptomatic; the virus persists in various tissues such as myeloid cells, vascular endothelium, and renal tissue [20]. The recent literature suggests that Herpesviridae reactivations are frequent in intensive care unit (ICU) patients with COVID-19. In particular, herpes simplex virus (HSV) and CMV re-activations have been reported at higher rates in these patients than those described in previous studies in critically ill patients without SARS-CoV-2 infection [22]. It is noteworthy that a significant proportion of the populations with high mortality rates due to SARS-CoV-2 infection, particularly in northern Italy and Spain, also show a high prevalence of CMV antibodies [23].

Evidence suggests that the incidence of herpes zoster, caused by the reactivation of the varicella zoster virus (VZV), has risen during the COVID-19 pandemic. This increase may be linked to the lymphopenia often seen with SARS-CoV-2 infection. Algaadi et al. recently reviewed several case reports on herpes zoster following COVID-19, concluding that there could be a potential causal relationship between the two conditions [24].

Also, the reactivation of the Epstein–Barr virus (EBV) has been commonly observed in patients with COVID-19, and some studies have suggested that it may be linked to increased morbidity and mortality. For example, Chen et al. found that EBV reactivation was prevalent among COVID-19 patients and was associated with symptoms like fever and heightened inflammation. Similarly, Xie et al. reported that 17 out of 128 COVID-19 patients (13.3%) exhibited signs of EBV reactivation. This group had higher mortality rates on days 14 and 28 compared to patients who did not experience EBV reactivation [25,26].

At the same time, there are several respiratory viruses that can cause co-infections, along with other respiratory pathogens, which is evidence that influenza viruses type A and B, or respiratory syncytial virus (RSV), could be present before or simultaneously with a SARS-CoV-2 infection. The coexistence of these infections can complicate the diagnosis and clinical management of patients [27]. During the COVID-19 pandemic, studies have reported cases of co-infections between SARS-CoV-2 and influenza viruses. These co-infections can influence the severity of the disease and have significant public health implications, especially during the flu season [27,28]. A study conducted by the Department of Microbiology at the Faculty of Medicine in Geneva showed that the prevalence of viral co-infections in SARS-CoV2-infected adults range from 3 to 21%, while in children, they vary between 5 and 19% [28,29].

Currently, there are numerous serological studies aiming to highlight the age at which populations become infected with CMV. These studies indicate that a portion of the pediatric population is infected perinatally, i.e., before the age of 6 months, while seroprevalence gradually increases with age. Interestingly, a higher prevalence is observed in females compared to males [30,31,32]. CMV infection profoundly impacts the human immune system, influencing the function of T cells, particularly T memory cells [33]. Regarding humoral immune responses, indicated by high levels of IgG antibodies to CMV, these have been associated with a greater likelihood of reactivation [34,35]. Similarly, we reported a patient with a recent history of a SARS-CoV-2 infection who subsequently developed multisystem inflammatory syndrome requiring both immunomodulatory therapy and high-dose steroids. Additionally, both SARS-CoV-2 infection and steroids could be triggers for a CMV infection. Thus, the patient developed CMV pneumonia, interpreting the infection as a reactivation, given the high titer of CMV IgG antibodies identified in the patient’s serum. This complicated the therapeutic decision due to the existing limitations in the pediatric population regarding the administration of specific antiviral therapy with intravenous ganciclovir.

The clinical manifestations of CMV pneumonia include dry cough, dyspnea, hypoxemia, and fever, which are nonspecific symptoms. Given the common manifestations shared with many other causes of pneumonia in the pediatric population, the diagnosis can be delayed [36,37,38,39]. A study conducted in China at the University Hospital in Suzhou over a period of three years on 1867 children under the age of 1 year who were hospitalized with lower respiratory tract infections due to Pertussis and CMV reactivation showed a high prevalence of CMV infection in immunocompetent patients [40]. Most patients received macrolides, but for 38 patients who were CMV-positive in plasma, intravenous ganciclovir was administered. These findings suggested that any viral or bacterial infection can be a trigger for latent CMV reactivation in an initially immunocompetent patient. Similarly, in our case, with the patient being immunocompetent, the SARS-CoV-2 infection and prolonged steroid therapy led to the reactivation of the CMV infection.

Regarding ganciclovir, opinions are divided, and there are no clear indications for its administration in immunocompetent patients with acquired or reactivated CMV infection in children under 12 years of age. However, the literature describes cases treated with ganciclovir at even younger ages than our patient for both pneumonia and gastrointestinal conditions. For example, the Pediatric Infectious Diseases Department at Johns Hopkins Medical Institutions in Baltimore and the Pediatric Infectious Diseases Department at the University of Texas reported two cases of CMV enterocolitis in two children, one 8 weeks old and the other 10 months old, who received intravenous ganciclovir treatment. Hematologic, hepatic, and renal parameters were monitored, and no notable adverse effects were observed [41]. Therefore, considering the risk–benefit balance of ganciclovir treatment, it was determined that the benefit far outweighs the possible adverse reactions, thus reducing long-term sequelae of this infection. Moreover, the benefits of ganciclovir treatment were also noticed in children with CMV pneumonia, even in immunocompetent children. Thus, a study performed on pediatric patients with pneumonia, which used ganciclovir for those with CMV detection, concluded that this antiviral drug is safe for this population. We must mention that the authors used ganciclovir in a dose of 10 mg/kg per 24 h for up to 14 days. Depending on the evolution, treatment continued for an additional 7 days at a dose of 5 mg/kg per 24 h, or until clinical and biological parameters were normalized (i.e., undetectable CMV DNA in plasma, normal CT images) [42].

## 4. Conclusions

MIS-C remains a challenge in pediatric pathology. Although it is a new entity, studies have been conducted and a series of cases have been reported from 2020 to the present. It is often confused with Kawasaki disease, which is not necessarily an error since the paraclinical investigations and therapeutic approach are similar, although in clinical practice, they are, of course, individualized based on the patient’s clinical and biological evolution.

On the other hand, latent CMV infection also remains relevant for clinical practice, especially in immunosuppressed individuals. Even though congenital infection, which is the most common, is well known, reinfection and reactivation are still topics of interest with respect to both adults and children. SARS-CoV-2 infection can cause CMV reactivation due to both the infection itself and the treatment administered to avoid dramatic events. Diagnosing CMV pneumonia is a challenge and should be considered in cases that are refractory to antibiotics.

## Data Availability

Data are available on request to the corresponding author due to privacy.
